# Mechanical Characterization of Multiwalled Carbon Nanotubes: Numerical Simulation Study

**DOI:** 10.3390/ma13194283

**Published:** 2020-09-25

**Authors:** Nataliya A. Sakharova, André F. G. Pereira, Jorge M. Antunes, José V. Fernandes

**Affiliations:** 1 Centre for Mechanical Engineering, Materials and Processes (CEMMPRE), DeptMech Engn, Univ Coimbra, Rua Luís Reis Santos, Pinhal de Marrocos, 3030-788 Coimbra, Portugal; andre.pereira@dem.uc.pt (A.F.G.P.); jorge.antunes@dem.uc.pt (J.M.A.); valdemar.fernandes@dem.uc.pt (J.V.F.); 2 Escola Superior de Tecnologia de Abrantes, Instituto Politécnico de Tomar, Rua 17 de Agosto de 1808, 2200-370 Abrantes, Portugal

**Keywords:** multiwalled carbon nanotubes, rigidity, Young’s and shear moduli, numerical simulation

## Abstract

The elastic properties of armchair and zigzag multiwalled carbon nanotubes were investigated under tensile, bending, and torsion loading conditions. A simplified finite element model of the multiwalled carbon nanotubes, without taking into account the van der Waals interactions between layers, was used to assess their tensile, bending, and torsional rigidities and, subsequently, Young’s and shear moduli. Relationships between the tensile rigidity and the squares of the diameters of the outer and inner layers in multiwalled carbon nanotubes, and between the bending and torsional rigidities with the fourth powers of the diameters of the outer and inner layers, were established. These relationships result in two consistent methods, one for assessment to the Young’s modulus of armchair and zigzag multiwalled carbon nanotubes, based on tensile and bending rigidities, and the other to evaluate shear modulus using tensile, bending, and torsional rigidities. This study provides a benchmark regarding the determination of the mechanical properties of nonchiral multiwalled carbon nanotubes by nanoscale continuum modeling approach.

## 1. Introduction

Multiwalled carbon nanotubes (MWCNTs) are structures composed of concentric single-walled carbon nanotubes (SWCNTs), the number of which can be comprised between 3 and 50. The interlayer spacing is commonly considered equal or close to the interlayer spacing of graphene, 0.34 nm, and the diameter of the MWCNTs can attain 100 nm, which contrasts with typical SWCNTs, whose diameters are between 0.7 and 2.0 nm [1].

Multiwalled and single-walled nanotubes have comparable properties, although the former have higher level of their commercialization [2] and some relative advantages [1]. For example, due to the multilayer structure of MWCNTs, the outer layers of nanotubes can protect the inner layers from external chemical interactions [3,4]. It is also worth to notice that, in last decade, MWCNTs with small diameters, containing only a few walls, have attracted the research attention (see, e.g., [5,6,7]), for making their functionalization possible, modifying only the outer layer of the MWCNTs and keeping the inner layers unchanged [8]. Multiwalled carbon nanotubes have been used for the development of novel electronic devices (see, e.g., [9,10,11,12]) and nanoelectromechanical systems [13,14,15,16].

Since the deformation influences the electron transport behavior of MWCNTs [17], the understanding of their mechanical properties is of great interest in the perspective of using nanotubes as constituents of electronic devices and nanoelectromechanical systems. From the point of view of reinforcement in composite materials, the MWCNTs, relatively abundant and widely commercialized, are good candidates for strengthening components. The correct design of these composites also demands knowing of the MWCNTs’ mechanical properties. MWCNTs’ exceptional mechanical properties, which can be advantageous for the enhancement of structural composites and the building of nanomechanical systems, have recently placed them in the focus of research interest.

Although plenty of experimental studies have been evidenced the outstanding mechanical properties of carbon nanotubes (CNTs), there is inconsistency in the experimental results reported in the literature, due to the complexity of characterizing nanomaterials at the atomic scale. As the experimental results concerning the mechanical properties of CNTs available in the literature show a wide scatter of their values, modelling and computer simulation studies have been established as the main approaches for the mechanical characterization of CNTs (both types of structure, SWCNTs and MWCNTs). There are three main groups of methodologies for modelling the mechanical behavior of CNTs: the atomistic, which comprises the molecular dynamic (MD) and ab initio approaches, the continuum mechanics (CM), and the nanoscale continuum mechanics (NCM). The NCM approach, which consists of replacing of each carbon–carbon (C–C) bond by a continuum element (such as a beam), whose behavior is described by the elasticity theory, has been recognized as the most appropriate for effective computational simulations and successfully used for simulation of the mechanical behavior of SWCNTs (see, [18,19,20]). Relatively few research efforts have been focused on building an adequate numerical model for the correct assessment to the mechanical properties of MWCNTs, which have more complex structure than SWCNTs. The main challenge when moving from SWCNTs to MWCNTs simulations is to take into account, in the simulation of the latter, the weak van der Waals (vdW) noncovalent force. This involves substantial modelling and computing efforts.

Although most studies on the evaluation of the MWCNTs elastic properties were carried out with simulations that use the NCM approach, some works employing other methodologies and even analytical approaches can be found in the literature. Hwang et al. [21] performed MD simulations resorting to empirical Tersoff three-body potential model, to study the mechanical behavior of double-walled and triple-walled carbon nanotubes (DWCNTs and TWCNTs). Santosh et al. [22] evaluated elastic moduli of DWCNTs using a generic force field approach in MD simulations. In these studies, the MD approach permitted to simulate covalent (carbon–carbon (C–C) bonds) and noncovalent (vdW forces) interactions between carbon atoms in the MWCNT structure. Tu and Ou-Yang [23] used the CM approach to describe the deformation behavior of single-walled and multiwalled CNTs. In their analytical study regarding the influence of the number of layers on the MWCNTs Young’s modulus, Tu and Ou-Yang [23] used the classic shell theory and considered MWCNT as a group of SWCNTs with a common central axis. The presence of van der Walls forces was not considered in this CM modelling. It was found that the Young’s modulus of MWCNTs with up to 100 SWCNTs layers is a function of the number of layers, strongly decreasing with the increase of this number in the MWCNT structure.

Among the studies that adopt the NCM approach, the first nonlinear truss rod model, taking into account the weak van der Waals noncovalent force, was developed by Li and Chou [24] in their study on the elastic behavior of MWCNTs with up to four layers under tension and torsion. Li and Chou [24] introduced complex mesh of the truss rods in addition to the beam element mesh for the simulation of each SWCNT composing the MWCNT. The studies that followed this work pursued to reduce the modelling efforts. With this purpose, Kalmakarov et al. [25] suggested a massless nonlinear spring element to simulate the van der Waals interactions. Later, a number of MWCNT’s models with up to five layers, employing spring elements to describe the van der Waals forces, were developed by Rahmandoust and Öchsner [26] and Ghavamian et al. [27,28]. Rahmandoust and Öchsner [26] showed that, in the case of uniaxial tensile test, the MWCNT’s models, taking into account the van der Waals force or not, lead to similar Young’s modulus results. However, for the case of the torsion test, a difference in shear modulus values of about 9.0% was reported between the results obtained with and without the van der Waals interactions. Consequently, Rahmandoust and Öchsner [26] determined that the modelling of the van der Waals interactions between atoms of neighbor layers (SWCNTs) of MWCNTs is not required in the case of tensile test. Rouhi et al. [29] reached a similar conclusion in their study on the elastic properties of double-walled and triple-walled nanotubes composed of carbon and boron nitride layers. Comparing the results of two finite element models generated with and without spring elements representing the van der Waals interactions, Rouhi et al. [29] found a small (less than 0.75%) difference in the Young’s modulus values.

In their FE model of DWCNTs and TWCNTs, Fan et al. [30] proposed the use of pressure between layers to model the van der Waals interactions, which helped to save the computing effort and led to results in reasonable agreement with those in literature. Aside from springs, other elements were employed to model the van der Waals forces, as in the work of Nahas and Abd-Rabou [31], in which beam elements were used to simulate the covalent carbon–carbon (C–C) bonds and the vdW force between layers, in DWCNTs and TWCNTs. In a recent study of Almagableh et al. [32], the finite element (FE) model of DWCNTs was developed, considering pseudorectangular beam elements, for simulating the covalent carbon–carbon (C–C) bonds, and nonlinear solid elements, whose stress–strain response was with the vdW interactions. This approach resulted in a complex nonlinear model, which permits accurate simulation of vdW interactions.

The aforementioned works provided the values of Young’s and shear moduli taken directly from numerical simulation analysis or calculated analytically. The numerical simulation studies related to MWCNTs rigidities, at our knowledge, are infrequent in the literature. In the work of Sakharova et al. [33], the tensile and bending rigidities were studied as a function of the outer layer diameter for nonchiral MWCNTs with up to 10 layers. In their work, Sakharova et al. [33] tested a simplified finite element model of MWCNTs without taking into account the van der Waals forces, but with boundary conditions that impose the simultaneous deformation of all the SWCNTs that constitute the MWCNT. A good agreement was found between the Young’s modulus values obtained from tensile and bending tests in this work [33], for nonchiral MWCNTs with up to 10 layers, with the results available in the literature.

Under certain conditions, nonlinear behavior was observed during the deformation of nanotubes. Although most work on this behavior is dealing with SWNCTs (see, e.g. (see, e.g., [34], where the buckling behavior of SWCNTs under bending was studied), several works were dedicated to MWCNTs, mainly the typical buckling mode, which makes the nanotube to curve when subjected to a compressive load greater than a certain critical load [35,36,37,38]. Chang et al. [36], based on a molecular mechanics model, pointed out two possible MWCNTs buckling modes: (i) the buckling occurring only on the outer wall (for MWCNTs with larger outer diameters) and (ii) the buckling occurring simultaneously on all individual layers (for MWCNTs with smaller outer diameters), depending on the values of the critical buckling strain, in each mode. They concluded that the van der Waals forces, between the layers of MWCNTs, have little effect on the critical buckling strain, in the case of DWCNT. Ru [37], using a simple shell model, also concluded that the van der Waals forces do not influence the critical buckling load of DWCNT. Yao et al. [39], who used continuum shell theory to investigate the buckling behavior of DWCNTs and MWCNTs up to five layers, under bending deformation, concluded that the greater the number of individual layers in the MWCNTs, the greater the required buckling load. Ghavamian and Öchsner [38] in their work on the buckling behavior of perfect and defective MWCNTs of up to five layers, based on the NCM approach, concluded that the buckling strength of MWCNTs significantly increases with increasing number of layers.

The present study aims to explore a simplified model of finite element MWCNTs without taking into account the van der Waals forces, in order to conduct a systematic study on the elastic properties of nonchiral (armchair and zigzag) MWCNTs, with up to 20 walls. The consideration of this number of walls brings the model closer to the real cases of MWCNTs. The work intends to contribute towards the understanding of the elastic behavior of MWCNTs under tensile, bending, and torsion loading conditions, focusing on the respective rigidities, and Young’s and shear moduli. The NCM approach, employing beam elements, was used to simulate individual layers, i.e., each SWCNT composing the MWCNTs [20,33]. A comprehensive study of the influence of the nanotube geometrical characteristics (the inner and outer layers diameters and the number of the constituent layers) on the tensile, bending, and torsional rigidities was carried out. Robust methods allow to easily characterize the elastic properties of nonchiral MWCNTs, whatever the diameter and the number of layers, without recourse to numerical simulation, are recommended for the first time. This can be useful, particularly for understanding and modelling the mechanical behavior of MWCNT reinforced composites. In addition, the present work provides a benchmark in relation to establishing the mechanical properties of nonchiral MWCNTs by nanoscale continuum models. In present analysis, the buckling effects were not considered, as the buckling did not occur for the strain values used.

## 2. Geometric Definition

The cylindrical structure of SWCNTs, whose surface is composed of carbon atoms in an hexagonal pattern, can be defined by the chiral vector, ***C_h_***, or the chiral angle, *θ,* between the chiral vector ***C_h_*** and the direction (*n*, 0) (see e.g., [40]):

(1) $$C_{h}=na_{1}+ma_{2}$$

(2) $$\theta ={sin}^{-1}\frac{\sqrt{3}m}{2\sqrt{n^{2}+nm+m^{2}}},$$

where (n,m) is a pair of integers that represents the lattice translation indices and ***a*_1_** and ***a*_2_** are the unit vectors of the graphene hexagonal lattice. The length of the unit vector ***a*** is defined as $a=\sqrt{3}a_{C-C}$ with the equilibrium carbon–carbon (C–C) covalent bond length $a_{C-C}=0.1421\;\mathrm{nm}$. In this context, zigzag (m=0, θ= 0°), armchair (n=m, θ= 30°), and chiral (n≠m, 0° < *θ* < 30°) nanotubes are the possible configurations of CNTs.

The SWCNT diameter is assessed as follows:

(3) $$D_{n}=\frac{a\sqrt{n^{2}+nm+m^{2}}}{\pi }.$$

Since MWCNTs comprise two or more coaxial SWCNTs (layers), their structure is characterized by a sequence of SWCNTs with pairs of chiral indices increasing from inside to outside so that the distance between layers is equal. For example, armchair MWCNT is described by the sequence $\left(n_{1},m_{1}\right)\left(n_{2},\;m_{2}\right)\ldots \left(n_{N},\;m_{N}\right)$, where *N* is the number of layers and n=m. Similarly, zigzag MWCNT is represented as $\left(n_{1},0\right)\left(n_{2},\;0\right)\ldots \left(n_{N},\;0\right)$, where *N* is the number of layers. The distance between layers in MWCNTs is generally considered similar to the interlayer spacing of graphene, 0.34 nm. Interlayer distance close to this value in the ranges of 0.32–0.35 nm and 0.342–0.375 nm was reported by Kharissova and Kharisov [41] and Kiang et al. [42], respectively.

## 3. Numerical Simulation and Analysis

### 3.1. Configurations and FE Modelling of MWCNT

The NCM approach that replaces the carbon–carbon bonds of CNT by equivalent beam elements was used for modelling each layer of MWCNTs, although, for the sake of simplicity, it does not consider van der Waals interactions between the layers. The finite element (FE) model uses the coordinates of the carbon atoms for generating the nodes and their suitable connection creates the beam elements. The links established between the interatomic potential energies of the molecular structure and the strain energies of the equivalent continuum structure, consisting of beams undergoing axial, bending, and torsional loading conditions, are at the basis of the application of continuum mechanics in the analysis of the mechanical behavior of CNT [24]. The input data for the FE model are given in Table 1.

The meshes of the MWCNTs structures, to be used in the FE analyses, were constructed using the CoNTub 1.0 software [43]. This code generates ASCII files, describing atom positions, which can be entered as input in available commercial and in-house FE codes to perform the simulation of mechanical tests. To convert the ASCII files, obtained from the CoNTub 1.0 software, into the format usable by the commercial FE code ABAQUS^®^, an in-house application previously developed, designated InterfaceNanotubes [20], was used. The geometrical characteristics of the armchair and zigzag MWCNTs used in the present FE analyses are summarized in Table 2 and Table 3, respectively. The multiwalled structures with different inner layer diameters and the same outer diameter were considered in order to clarify the influence of the geometrical characteristics of inner SWCNT on MWCNTs mechanical properties. In case of armchair MWCNTs, the structures with different inner layers, (10, 10) SWCNT with diameter $D_{in}$ = 1.356 nm, and (35, 35) SWCNT with diameter $D_{in}$ = 4.746 nm were studied (see, Table 2). The outer diameter for both cases was $D_{out}=14.238\;\mathrm{nm}$. The sequences of pairs of chiral indices for armchair MWCNT structures are given by (10+5(N−1), 10+5(N−1)), 2≤N≤20, and (35+5(N−1), 35+5(N−1)), 2≤N≤15, where N is the number of layers. In case of zigzag MWCNTs, the inner layers of the multiwalled structures under study were (14, 0) SWCNT with $D_{in}$ = 1.096 nm and (59, 0) SWCNTs with $D_{in}$ = 4.619 nm, and the outer layers had the same diameter of $D_{out}$ = 14.483 nm (see, Table 3). The sequences of pairs of chiral indices for zigzag MWCNTs are given by (14+9(N−1), 0), 2≤N≤20, and (59+9(N−1), 0), 2≤N≤15. The interlayer spacing, $d_{int}$, of these MWCNT structures is close to the interlayer spacing of graphene, 0.34 nm, i.e., 0.339 nm and 0.352 nm for armchair and zigzag MWCNTs, respectively. The length of the nanotubes used in the numerical simulations was about 30 times bigger than the outer diameter, such that the mechanical behavior can be independent of the length.

### 3.2. Loading Conditions

**Figure 1:**
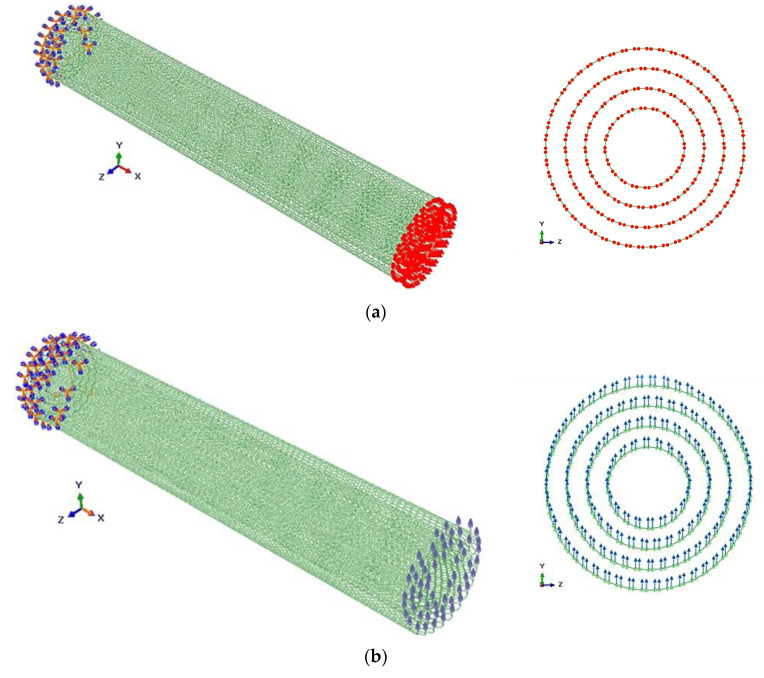

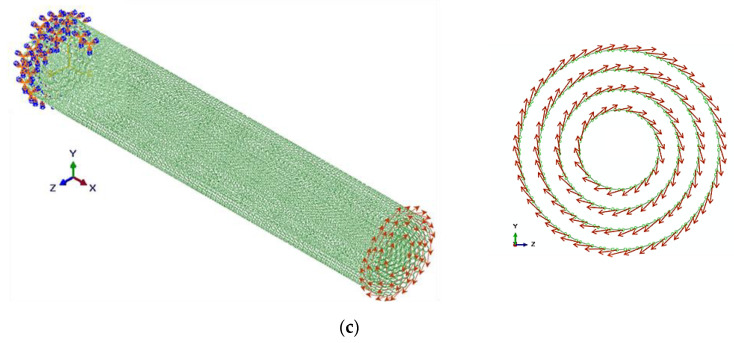
Loading and boundary conditions for the (10,10) (15,15) (20,20) (25,25) armchair multiwalled carbon nanotube (MWCNT): (**a**) tension, (**b**) bending, and (**c**) torsion.

The mechanical behavior of MWCNTs was studied by numerical simulation using the commercial FE code ABAQUS^®^ of conventional tensile, bending, and torsion tests. The boundary and loading conditions are shown in Figure 1.

In the tensile test, an axial displacement, $u_{x}$, is applied to all nodes of one MWCNT end, leaving the other end fixed. The tensile rigidity of the nanotube, EA, is determined as:

(4) $$EA=\frac{F_{x}L}{u_{x}}\;,$$

where L is the nanotube length and $F_{x}$ is an axial force, taken from the FE analysis.

In the bending test, a transverse displacement, $u_{y}$, is applied to all nodes of one MWCNT end, leaving the other end fixed. The bending rigidity of the nanotube, EI, is determined as:

(5) $$EI=\frac{F_{y}L^{3}}{3u_{y}}\;,$$

where $F_{y}$ is the transverse force, taken from the FE analysis.

In the torsion test, a torsional moment, T, is applied at one MWCNT end, forcing the atoms of that end to the same rotation and leaving the atoms of the other end fixed. The torsional rigidity, GJ, is determined as:

(6) $$GJ=\frac{TL}{\varphi }\;,$$

where φ is the twist angle, taken from the FE analysis.

### 3.3. Elastic Moduli of MWCNTs

The Young’s modulus of MWCNTs was calculated using the following expression that takes into account the rigidity in tension, EA, calculated from Equation (4):

(7) $$E=\frac{EA}{A},$$

where A is the cross-sectional area.

In addition, the Young’s modulus of MWCNTs was calculated taking into account the rigidity in bending, EI, calculated from Equation (5):

(8) $$E=\frac{EI}{I}.$$

The shear modulus of MWCNTs was calculated using the following expression that takes into account the rigidity in torsion, GJ, calculated from Equation (5):

(9) $$G=\frac{GJ}{J},$$

where J is the polar moment of inertia.

**Figure 2:**
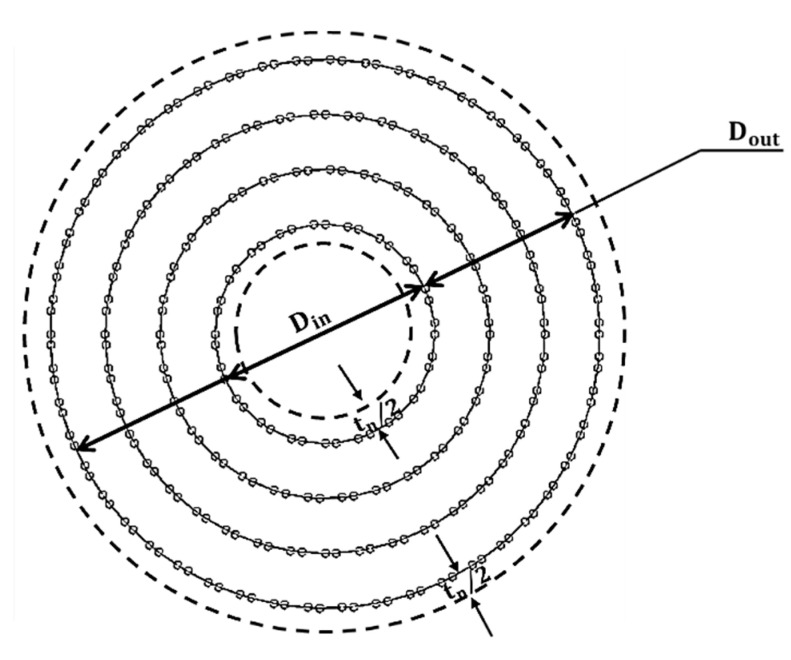
Schematic representations of the MWCNT profile.

MWCNTs with the inner layer diameter, $D_{in}$, the outer layer diameter, $D_{out}$, and the thickness of layers, $t_{n}$ (see Figure 2), have the cross-sectional area, the moment of inertia, and the polar moment of inertia of the equivalent hollow cylinder given by, respectively:

(10) $$A=\frac{\pi }{4}\left[{\left(D_{out}+t_{n}\right)}^{2}-{\left(D_{in}-t_{n}\right)}^{2}\right],$$

(11) $$I=\frac{\pi }{64}\left[{\left(D_{out}+t_{n}\right)}^{4}-{\left(D_{in}-t_{n}\right)}^{4}\right],$$

(12) $$J=\frac{\pi }{32}\left[{\left(D_{out}+t_{n}\right)}^{4}-{\left(D_{in}-t_{n}\right)}^{4}\right].$$

Assigning $\bar{D}$ = ($D_{in}+D_{out})/2$ as the average diameter of MWCNT and $∆D=D_{out}-D_{in}$ as the difference between the diameters of the outermost and innermost layers, the Equations (10)–(12) can be modified as follows:

(13) $$A=\frac{\pi }{2}\bar{D}\left(∆D+2t_{n}\right),$$

(14) $$I=\frac{\pi }{16}\bar{D}\left(∆D+2t_{n}\right)\cdot \left[2{\bar{D}}^{2}-D_{out}D_{in}+t_{n}\left(∆D+t_{n}\right)\right],$$

(15) $$J=\frac{\pi }{8}\bar{D}\left(∆D+2t_{n}\right)\cdot \left[2{\bar{D}}^{2}-D_{out}D_{in}+t_{n}\left(∆D+t_{n}\right)\right].$$

Adopting an analysis similar to that followed for in a previous study for SWCNTs [20], using Equations (13) and (14) is now possible to write:

(16) $$\begin{array}{c} \frac{EI}{EA}=\frac{1}{8}\left[2{\bar{D}}^{2}-D_{out}D_{in}+t_{n}\left(∆D+t_{n}\right)\right]\Rightarrow \\ \bar{D}=\frac{1}{\sqrt{2}}\sqrt{8\left(\frac{EI}{EA}\right)+\left[D_{out}D_{in}-t_{n}\left(∆D+t_{n}\right)\right]}. \end{array}$$

Consequently, the Young’s modulus of MWCNTs can be calculated from Equations (7), (13), and (16):

(17) $$E=\frac{EA}{A}=\frac{EA}{\pi \left(∆D+2t_{n}\right)\;\sqrt{\left(\frac{EI}{EA}\right)+\frac{1}{8}\left[D_{out}D_{in}-t_{n}\left(∆D+t_{n}\right)\right]\;}\;}.$$

The shear modulus of MWCNTs can be calculated from Equations (8), (14), and (15):

(18) $$G=\frac{GJ}{J}=\frac{GJ}{2\pi \left(\frac{EI}{EA}\right)\left(∆D+2t_{n}\right)\;\sqrt{\left(\frac{EI}{EA}\right)+\frac{1}{8}\left[D_{out}D_{in}-t_{n}\left(∆D+t_{n}\right)\right]\;}\;}.$$

The value of the nanotube wall thickness to be considered is $t_{n}=0.34\;\mathrm{nm}$, as very commonly used [18,20,26,27].

## 4. Results and Discussion

### 4.1. Rigidities of MWCNTs

**Figure 3:**
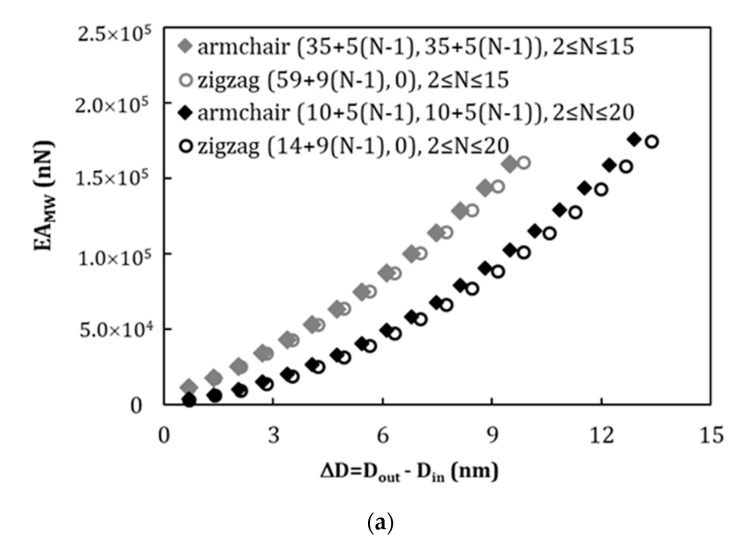

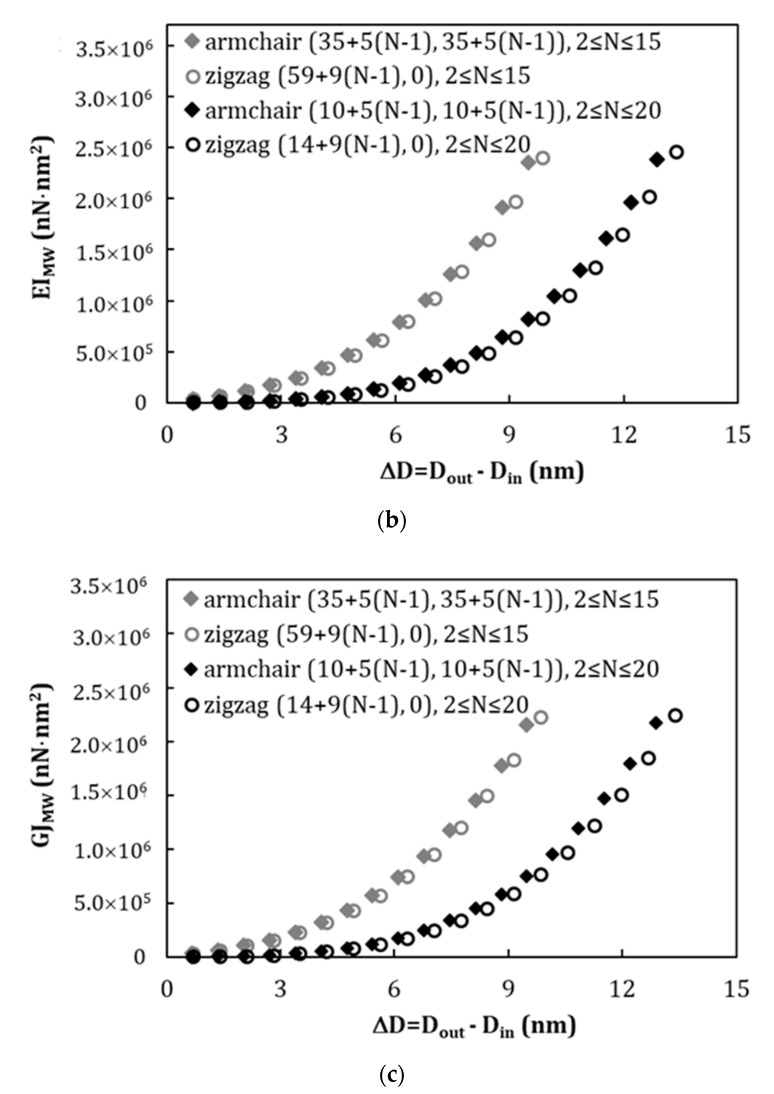
Evolution of rigidity as a function of the difference between the diameters of the outer and inner layers, ∆D, for armchair and zigzag MWCNTs, in: (**a**) tension, $EA_{MW}$, (**b**) bending, $EI_{MW}$, and (**c**) torsion, $GJ_{SW}$.

The values of the tensile, $EA_{MW}$, bending, $EI_{MW}$, and torsional, $GJ_{MW}$, rigidities of the MWCNTs, numerically obtained and using Equations (4)–(6), are represented as a function of the difference between the outer and the inner layers diameters, $∆D=D_{out}-D_{in}$, in the Figure 3a–c, respectively. For each nanotube type, the evolutions of tensile rigidity, $EA_{MW}$, are clearly separated, depending on the diameter of the inner layer of the MWCNTs: (i) for the smallest inner layer, $D_{in}$ = 1.356 nm for (10+5(N−1), 10+5(N−1)) armchair and $D_{in}$ = 1.096 nm for (14+9(N−1), 0) zigzag structures and (ii) for the largest inner layer diameter, $D_{in}$ = 4.746 nm for (35+5(N−1), 35+5(N−1)) armchair and $D_{in}$ = 4.619 nm for (59+9(N−1), 0) zigzag MWCNTs. The same is true for the evolutions of the bending, $EI_{MW}$, and torsional, $GJ_{SW}$, rigidities with $∆D=D_{out}-D_{in}$.

**Figure 4:**
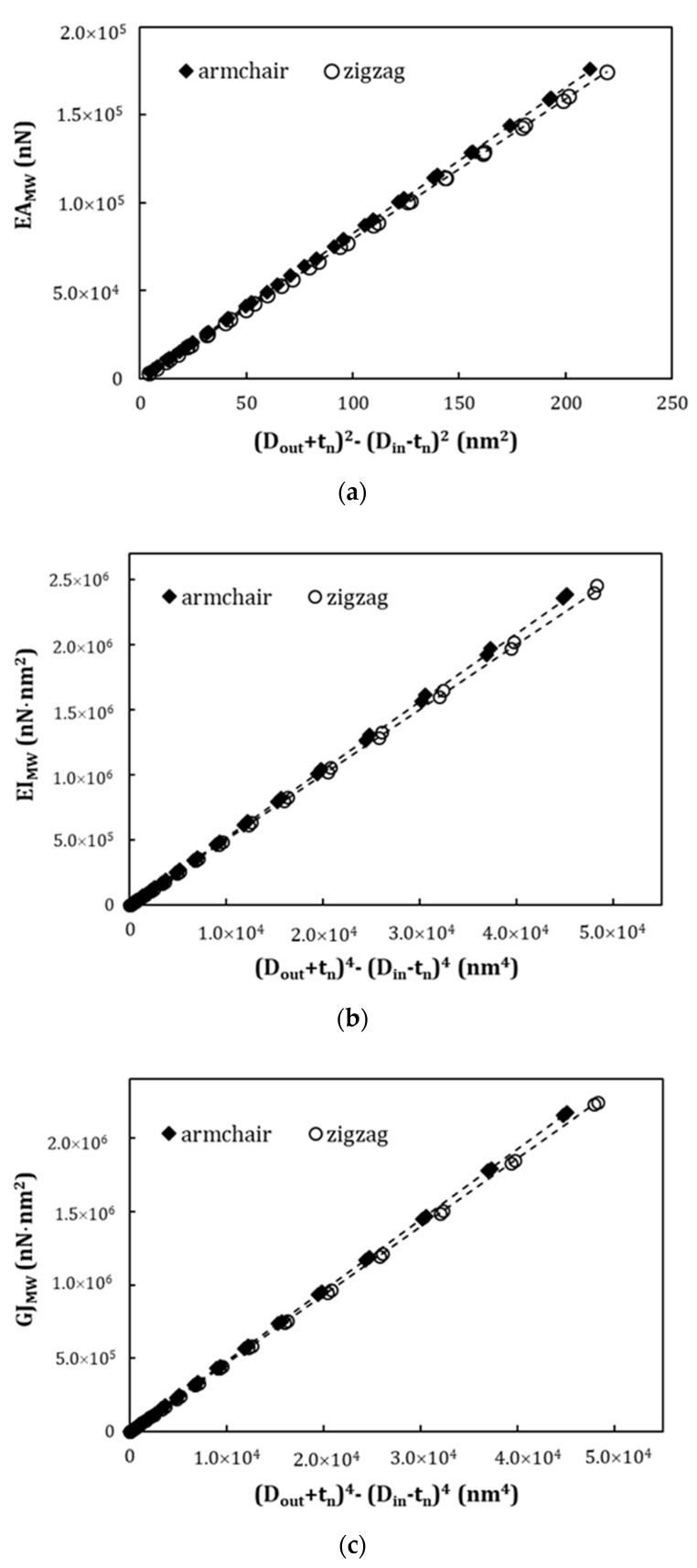
Evolutions of rigidities as a function of: (**a**) ${\left(D_{out}+t_{n}\right)}^{2}-{\left(D_{in}-t_{n}\right)}^{2}$, for the tensile rigidity, $EA_{MW}$; (**b**) ${\left(D_{out}+t_{n}\right)}^{4}-{\left(D_{in}-t_{n}\right)}^{4}$ for bending rigidity, $EI_{MW}$; and (**c**) ${\left(D_{out}+t_{n}\right)}^{4}-{\left(D_{in}-t_{n}\right)}^{4}$ for torsional rigidity, $GJ_{SW}$. The results are represented by symbols and fitting trends by lines.

In order to clarify the trends shown in Figure 3a–c, the values of the tensile rigidity, $EA_{MW}$, are plotted as a function of ${\left(D_{out}+t_{n}\right)}^{2}-{\left(D_{in}-t_{n}\right)}^{2}$, and the values of the bending, $EI_{MW}$, and torsional, $GJ_{SW}$, rigidities are plotted as a function of ${\left(D_{out}+t_{n}\right)}^{4}-{\left(D_{in}-t_{n}\right)}^{4}$, in Figure 4a–c. These plots are inspired by the expressions 10, 11, and 12 of the area and the moments of inertia of the MWCNTs. Figure 4a–c show that, for this representation, for each type of nanotube, the results follow the same straight line, regardless of the diameter of the inner nanotube. Furthermore, the results are only slightly influenced by the type of nanotube, i.e., armchair or zigzag MWCNTs. This small difference in the rigidity behavior of the two types of MWCNTs can be attributed to the different interlayer spacing of these structures: armchair ($d_{int}=0.339\;\mathrm{nm}$) and zigzag ($d_{int}=0.352\;\mathrm{nm}$). The straight lines in Figure 4a–c can be expressed as follows:

(19) $$EA_{MW}=\alpha_{MW}\left[{\left(D_{out}+t_{n}\right)}^{2}-{\left(D_{in}-t_{n}\right)}^{2}\right],$$

(20) $$EI_{MW}=\beta_{MW}\left[{\left(D_{out}+t_{n}\right)}^{4}-{\left(D_{in}-t_{n}\right)}^{4}\right],$$

(21) $$GJ_{MW}=\gamma_{MW}\left[{\left(D_{out}+t_{n}\right)}^{4}-{\left(D_{in}-t_{n}\right)}^{4}\right].$$

The fitting parameters for armchair and zigzag MWCNTs are given in Table 4. The mean difference between the values of the $EA_{MW}$, $EI_{MW}$, and $GJ_{SW}$ rigidities calculated with Equations (19)–(21) and the values obtained directly from FE analysis are, respectively, 0.10%, 1.23%, and 0.45% for armchair nanotubes and 0.35%, 0.97%, and 0.31% for zigzag nanotubes.

### 4.2. Young’s and Shear Moduli of MWCNTs

The Young’s and shear moduli of the (10+5(N−1), 10+5(N−1)), 2≤N≤20, armchair and (14+9(N−1), 0), 2≤N≤20, zigzag MWCNTs structures are analyzed in this subsection.

The MWCNTs Young’s modulus values can be calculated by Equation (7) or Equation (8) using the results of the numerical tensile and bending tests, respectively, and Equation (17), which uses the results of numerical tensile and bending tests. The values of the MWCNTs shear modulus can be calculated by Equation (9), using only the numerical torsional test results, and Equation (18), which uses the results of numerical tensile, bending, and torsional tests.

Equation (17) and relationships (19)–(21) as well as the knowledge of the values of the parameters $\alpha_{MW}$, $\beta_{MW}$, and $\gamma_{MW}$ in Table 4 allow the easy determination of the Young’s modulus of the MWCNTs, as a function of the outer layer diameter, $D_{out}$, and the inner layer diameter, $D_{in}$, without resorting to the numerical simulation as follows:

(22) $$E\;=\frac{\alpha_{MW}\left(D_{out}+D_{in}\right)}{\pi \;\sqrt{\frac{\beta_{MW}}{\alpha_{MW}}\left[D_{out}^{2}+D_{in}^{2}+2t_{n}\left(∆D+t_{n}\right)\right]+\frac{1}{8}\left[D_{out}D_{in}-t_{n}\left(∆D+t_{n}\right)\right]}\;}.$$

In the same way, but using the Equation (18) and relationships (19)–(21), the shear modulus of the MWCNTs can be calculated as follows:

(23) $$G=\frac{\gamma_{MW}\left(D_{out}+D_{in}\right)}{2\pi \left(\frac{\beta_{MW}}{\alpha_{MW}}\right)\sqrt{\frac{\beta_{MW}}{\alpha_{MW}}\left[D_{out}^{2}+D_{in}^{2}+2t_{n}\left(∆D+t_{n}\right)\right]+\frac{1}{8}\left[D_{out}D_{in}-t_{n}\left(∆D+t_{n}\right)\right]}\;}.$$

**Figure 5:**
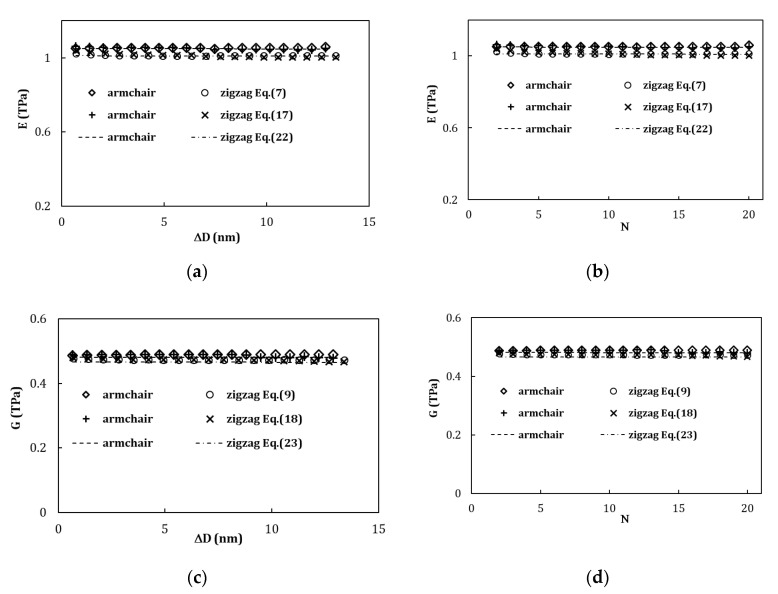
Evolutions of the Young’s modulus of MWCNTs with: (**a**) the difference between outer and inner layers diameters, ∆D, and (**b**) the number of layers, N, constituting the MWCNT and evolutions of the shear modulus of MWCNTs with: (**c**) ∆D and (**d**) N. The Young’s moduli of MWCNTs were evaluated by Equations (7) and (17), from the numerical simulations results, and analytically by Equation (22). The shear moduli of MWCNTs were evaluated by Equations (9) and (18), from the numerical simulations results, and analytically by Equation (23).

Figure 5 compares the Young’s and shear modulus results as a function of the difference between outer and inner layers diameters, ∆D (Figure 5a,c), and the number of layers, N, (Figure 5b,d) in the MWCNT structure, for armchair and zigzag nanotubes. The Young’s and shear modulus values were obtained by Equation (7) (Equation (8) leads to results with a relative difference of 0.9%, on average) and (17) and by Equations (9) and (18), respectively, using numerical simulation results. The evolutions of the E and G values, calculated analytically by Equations (22) and (23), respectively, are also plotted for armchair and zigzag structures. 

For both types, armchair and zigzag MWCNTs, the evolutions of the Young’s modulus, E, and shear modulus, G, are similar, regardless of the equation (used for its calculation (Equation (7) or Equation (17), for the Young’s modulus, and Equation (9) or Equation (18), for the shear modulus). Moreover, Equation (22) permits accurate analytical prediction of the value of the Young’s modulus of MWCNTs as well as Equation (23) for the shear modulus value.

The mean difference between the Young’s moduli calculated, e.g., from Equation (17), using numerical results, and those evaluated with Equation (22) is 0.32% and 0.53% for structures of armchair and zigzag, respectively. For the shear modulus, the mean difference between its values calculated by Equation (18) and those evaluated with Equation (23) is 1.55% and 1.62% for armchair and zigzag structures, respectively.

It can be seen from Figure 5 that the Young’s modulus values of the armchair structure are slightly higher (in average 3.8%) than those for the zigzag structure. In the case of shear modulus, its values for armchair structure are in average 1.5% higher than those for the zigzag structure. The Young’s and shear modulus values of the armchair MWCNTs is about the same regardless the value of $∆D=D_{out}-D_{in}$ (Figure 5a,c) or the number of layers, N (Figure 5b,d) constituting the MWCNT. In the case of zigzag MWCNTs, a slight decrease of the Young’s modulus value (at about 2.50%) and shear modulus value (at about 2.08%) is observed when the difference between outer and inner layers’ diameters (Figure 5a,c) or the number of layers (Figure 5b,d) increase. As pointed out above in relation to rigidity, the differences between the Young’s modulus of the armchair and zigzag MWCNTs are also certainly related with the different interlayer spacing for armchair and zigzag structures. The same is true for the differences between the shear moduli of the armchair and zigzag MWCNTs.

**Figure 6:**
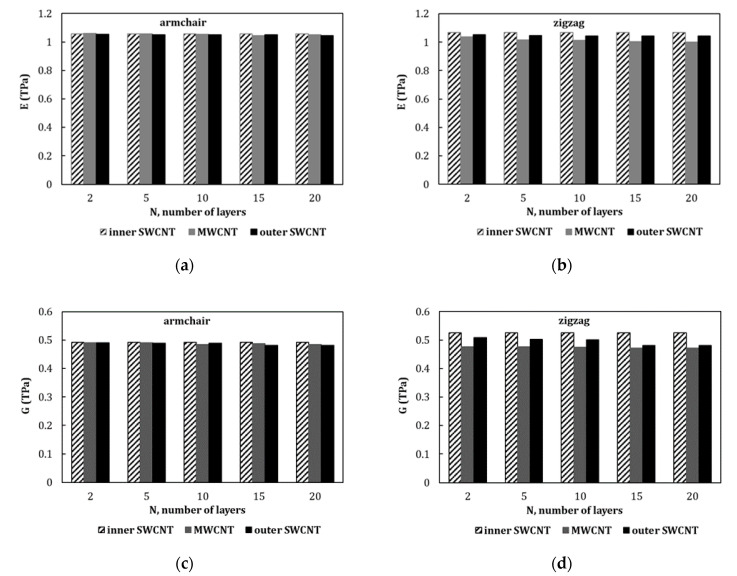
Young’s moduli of MWCNTs compared with the Young’s moduli of the innermost and outermost constituent SWCNTs for nanotubes structures: (**a**) armchair and (**b**) zigzag; shear moduli of MWCNTs compared with the shear moduli of the innermost and outermost constituent SWCNTs for: (**c**) armchair and (**d**) zigzag structures.

Figure 6 compares the Young’s modulus of the MWCNTs with the Young’s moduli of SWCNTs corresponding to the inner and outer constituent layers, for selected armchair (Figure 6a) and zigzag (Figure 6b) MWCNTs with 2, 5, 10, 15, and 20 layers. In this figure, the comparison of the MWCNTs’ shear modulus with the shear moduli of the inner and outer constituent SWCNTs is also shown for armchair (Figure 6c) and zigzag (Figure 6d) structures. The Young’s and shear modulus values obtained by Equations (17) and (18), respectively, are used. The Young’s modulus values for the armchair MWCNTs are very close to the values of E obtained for the inner and outer layers. The Young’s modulus values for zigzag MWCNTs are lower than the Young’s moduli of the inner and outer constituent layers. The same trends are observed for the shear modulus values.

In order to further test the methodology for analytical evaluation of the Young’s and shear moduli of MWCNTs (Equations (22) and (23)), two sets of MWCNTs (armchair and zigzag), for which the innermost nanotube layer is still smaller in diameter than those analyzed so far, were considered. The configurations for both sets, armchair and zigzag, MWCNTs are shown in Table 5.

**Figure 7:**
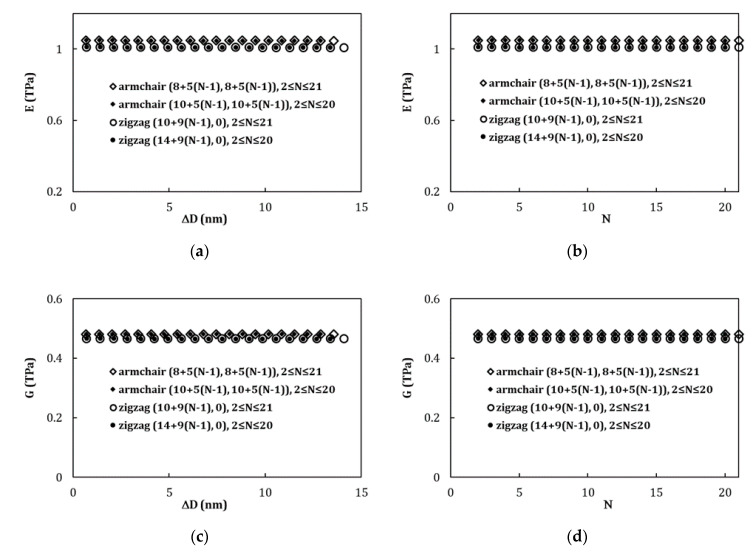
Evolutions of Young’s modulus with: (**a**) the difference between outer and inner layers diameters, ∆D, and (**b**) the number of layers, N, constituting the MWCNT; evolutions of the shear modulus of MWCNTs with: (**c**) ∆D and (**d**) N, for (8+5(N−1), 8+5(N−1)) and (10+5(N−1), 10+5(N−1)) armchair and (10+9(N−1), 0) and (14+9(N−1), 0) zigzag MWCNTs. The Young’s and shear modulus values were calculated by Equations (22) and (23), respectively.

Figure 7 compares the Young’s and shear moduli values obtained by Equations (22) and (23), in function of the difference between outer and inner layers diameters, ∆D, (Figure 7a,c), and the number of layers, N, (Figure 7b,d) in the MWCNTs structure, for (8+5(N−1), 8+5(N−1)) (Table 5) and (10+5(N−1), 10+5(N−1)) (Table 2) armchair and (10+9(N−1), 0) (Table 5) and (14+9(N−1), 0) (Table 3) zigzag MWCNTs. The Young’s modulus values obtained are similar for two sets of armchair MWCNTs, as well as for two sets of zigzag MWCNTs. The same is true for shear modulus values.

### 4.3. Comparison with Literature Results

Table 6 summarizes the current elastic moduli results of MWCNTs and those from literature, which include numerical and experimental results.

With regard to experimental evaluations, in the work of Treacy et al. [46], the MWCNT Young’s modulus was obtained by measuring the amplitude of the nanotube intrinsic thermal vibrations by transition electron microscopy (TEM). Kashyap and Patil [47] evaluated the Young’s modulus of MWCNT from TEM bright field image of CNT/Al composite.

The Young’s shear moduli were calculated from the numerical results of the conventional tensile [21,24,25,27,30,31,32] and torsion [22,24,25,28,30] tests, respectively, using the respective definitions from the classical theory of elasticity. Santosh et al. [22] evaluated the MWCNTs’ Young’s modulus from the numerical results of conventional compression test. With regard to the boundary conditions, the simulation of the MWCNT’s tensile test, in the works of [24,25,32], was achieved by subjecting all nodes at one end to the same axial force, while all nodes at the other end were fixed. In the simulation of torsion tests, Kalmakarov et al. [25], Fan et al. [30], and Santosh et al. [22] applied a torsional moment to all end nodes of multiwalled nanotube, but in the study of Li and Chou [24] only the outer layer of MWCNT was subjected to torsion. Ghavamian et al. [27,28], in tensile and torsion tests, Nahas and Abd-Rabou [31] and Hwang et al. [21], in tensile tests, and Santosh et al. [22], in compression tests, applied displacements, instead of forces or moments, to all nodes at one end of the MWCNT, leaving the other end fixed.

**Figure 8:**
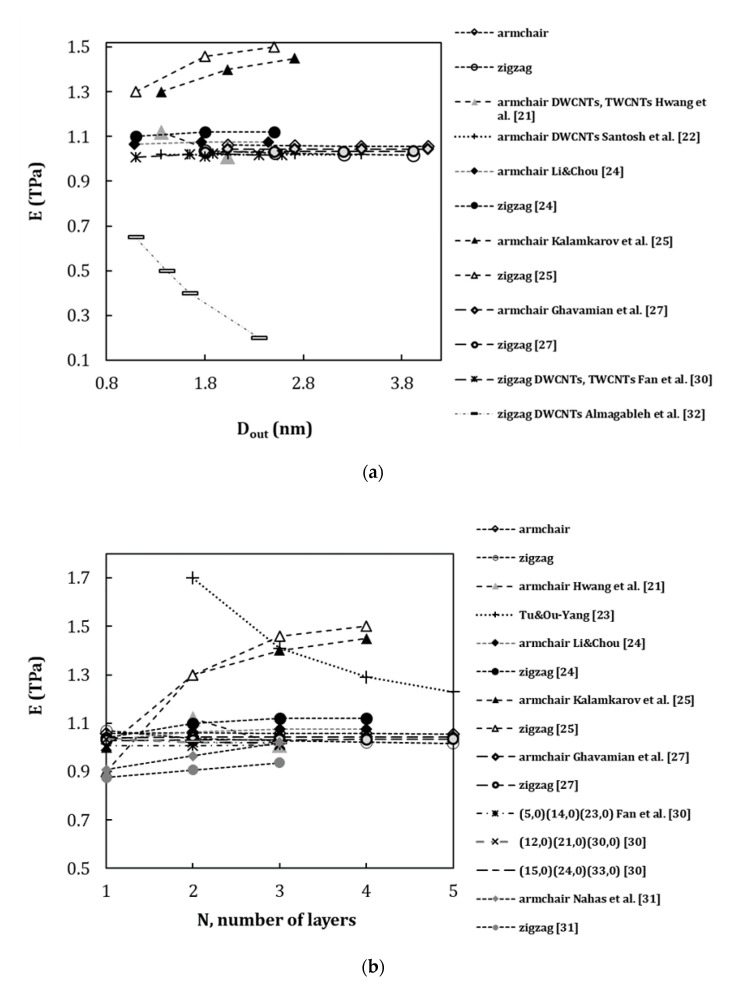
Comparison of the current results of the Young’s modulus with those reported in the literature as a function of: (**a**) outer layer diameter, $D_{out}$, and (**b**) the number of layers, N, of the constituting MWCNT.

In order to facilitate the comparison of the current results with those available in the literature, the Young’s and shear moduli were represented as a function of the outer layer diameter, $D_{out}$, of the MWCNT and the number of layers, N, constituting the MWCNT structure, as shown in Figure 8 (please see, designations in Table 6). MWCNTs with $D_{out}$ up to 4.068 nm were considered. This diameter corresponds to multiwalled structures containing up to five layers. The results from the works [21,23,24,25,27,30,31], which permit appropriate comparison of the Young’s modulus evolution with N, were considered in the figures.

Most aforementioned studies share the same modelling approach for the simulation of the MWCNT structure, i.e., a NCM approach employing 3D beam elements [24,25,27,30,31,32], although Almagableh et al. [32] used rectangular cross-section beams instead of circular ones. Regarding the simulation of the noncovalent van der Waals interactions between layers, truss rod elements [24], spring elements [25,27,31], nonlinear solid elements [32], and beam elements [31] were used. The model proposed by Tu and Ou-Yang [23] does not take into account the van der Waals forces, and in the works of Hwang et al. [21] and Santosh et al. [22], the vdW forces were modelled in a frame of MD approaches used.

Some authors [22,24,27,30] pointed out that Young’s modulus of MWCNTs did not change significantly with the increase of the outer layer diameter, $D_{out}$, and the number of layers, N, composing the MWCNT structure. A substantial increase of the Young’s modulus with the increase of the outer layer diameter or the number of layers was reported by Kalmakarov et al. [25]. Nahas and Ab-Rabou [31] noted slight increase of the Young’s modulus values with the number of layers for DWCNTs and TWCNTs. A considerable decrease of the Young’s modulus with the increase of the outer layer diameter was reported by Almagableh et al. [32] for zigzag DWCNTs, and Hwang et al. [21] reported slight reduction of the Young’s modulus upon transition from armchair DWCNT (N=2) to TWCNT (N=3). Tu and Ou-Yang [23] predicted a substantial reduction in Young’s modulus (from 4.70 TPa for SWCNT to 1.05 TPa for MWCNT with N=100) with the increase of the number of layers in the MWCNT structure. Some authors [24,30,31] pointed out that Young’s modulus of MWCNTs is slightly higher than that SWCNTs, but Young’s modulus values for MWCNTs, which are very close to the values obtained for SWCNTs constituting the MWCNT, were also reported [27].

The current results show particularly good agreement with the results of: (i) Fan et al. [30] (Figure 8a,b), for zigzag MWCNTs; (ii) Ghavamian et al. [27] (Figure 8a,b), for armchair and zigzag MWCNTs, where the spring elements for simulation of the vdW interactions were considered; (iii) Li and Chou [24] (Figure 8a,b), for armchair MWCNTs, who used the truss rod elements for simulation of the van der Waals forces; (iv) Santosh et al. [22] (Figure 8b) for armchair DWCNTs; and (v) Hwang et al. [21] (Figure 8a,b), for armchair DWCNTs and TWCNTs, where both covalent and vdW interactions between carbon atoms were modelled with recourse of MD approach. The smallest difference of 0.79% occurs for the Young’s modulus calculation performed by Fan et al. [30] for (15, 0)(24, 0)(33, 0) zigzag TWCNTs with $D_{out}=2.584\;\mathrm{nm}$. Differences of 1.39% and 1.62% occur for the results of Ghavamian et al. [27] for armchair and zigzag MWCNTs, respectively. The comparison with the results reported by Li and Chou [24] shows differences of 1.41% and 8.48% for armchair and zigzag MWCNTs, respectively. The Young’s modulus values obtained by Nahas and Abd-Rabou [31] (Figure 8b) show differences of 3.4% and 9.5% for armchair and zigzag MWCNTs, respectively, when compared with the current results. The Young’s modulus calculated by Nahas and Abd-Rabou [31] is also lower than those obtained in the other studies [21,22,23,24,25,27,30]. Differences of 3.54% and 4.82% were observed with the results of Santosh et al. [22] for armchair DWCNTs with $D_{out}$ up to 3.390 nm and Hwang et al. [21] for armchair TWCNTs with $D_{out}=2.034\;\mathrm{nm}$, respectively.

Substantial differences (36.9% for armchair and 46.0% for zigzag MWCNTs with N=4) were found with the Young’s modulus results predicted by Kalamkarov et al. [25] (Figure 8b). The biggest differences, in the range of 39.2% for (5, 0)(14, 0) DWCNTs with $D_{out}=1.096\;\mathrm{nm}$ to 50.70% for (21, 0)(30,0) DWCNT with $D_{out}=2.349\;\mathrm{nm}$, were observed with the results obtained by Almagableh et al. [32] (Figure 8a) and from 60.17% (for MWCNT with N=2) to 16.45% (for MWCNT with N=5) with the Young’s modulus values calculated by Tu and Ou-Yang [23] (Figure 8b).

**Figure 9:**
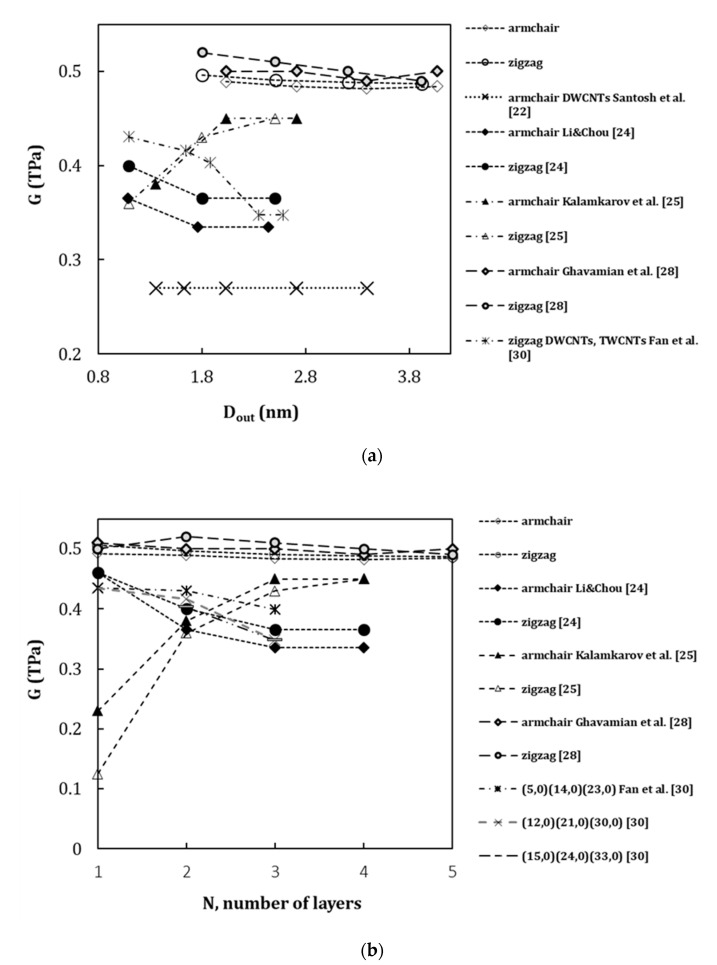
Comparison of the current results of the shear modulus with those reported in the literature as a function of: (**a**) outer layer diameter, $D_{out}$, and (**b**) the number of layers, N, of the constituting MWCNT.

Figure 9 compares current results of the shear modulus in function of the outer layer diameter, $D_{out}$ (Figure 9a) and the number of layers, N, constituting the MWCNTs (Figure 9b), with the results available in the literature (see, Table 6). As in the case of the Young’s modulus, MWCNTs with $D_{out}$ up to 4.068 nm, which corresponds to up to five layers in the structure, were considered [22,24,25,28,30].

Kalmakarov et al. [25] observed a substantial increase of the shear modulus with the increase of the outer layer diameter or the number of layers, and Santosh et al. [22] and Ghavamian et al. [28] pointed out an almost constant shear modulus with the increase of $D_{out}$ and N. Li and Chou [24] and Fan et al. [30] reported lower shear modulus values for MWCNT than for SWCNTs, and in both studies, a decreasing trend was observed for the shear modulus with the increase of the outer layer diameter or the number of layers.

When compared with current results of the shear modulus, the smallest differences of 2.85% and 2.57% occur for the results of Ghavamian et al. [28] for armchair and zigzag MWCNTs, respectively. The comparison with the results reported by Li and Chou [24] shows substantial differences of 23.27% and 19.85% for armchair and zigzag MWCNTs, respectively. The differences of 22.28% for armchair and 30.75% for zigzag structures with current results are observed for shear modulus values calculated by Kalmakarov et al. [25]. The shear modulus obtained by Fan et al. [30] for (15,0)(24,0)(33,0) TWCNT with $D_{out}=2.584\;\mathrm{nm}$ shows the difference of 29.18% when compared with current results. The biggest difference of 44.42% is observed with the results of Santosh et al. [22] evaluated for armchair DWCNTs. The shear modulus calculated by Santosh et al. [22] is also lower than those obtained in the other studies [24,25,28,31].

In summary, the current results show that reliable values of the Young’s and shear moduli were obtained when compared with several of the literature results.

## 5. Conclusions

A simplified finite element model of multiwalled carbon nanotubes, disregarding the van der Waals forces that act between layers, was used in order to carry out a systematic evaluation of the tensile, bending and torsional rigidities and, subsequently, of the Young and shear moduli of nonchiral (armchair and zigzag) MWCNT structures with up to 20 constituent SWCNTs, which approaches the present modelling to actual cases of MWCNTs.

The main conclusions of this comprehensive study are as follows:

- A linear relationship was established between the tensile rigidity of MWCNTs and the squares of the diameters of the outer and inner layers; Also, a linear relationship relates the bending and torsional rigidities of MWCNTs with the fourth powers of the diameters of the outer and inner layers.
- These linear relationships allow to obtain equations for easy analytical evaluation of the Young’s and shear moduli of armchair and zigzag MWCNT structures.
- Equations (22) and (23) offer two robust methods suitable for low-resource assessment to the Young’s and shear moduli of armchair and zigzag MWCNTs. They allow easily characterize the elastic moduli of nonchiral MWCNTs, whatever their inner and outer diameters, and the number of walls. This can be particularly useful, when understanding and modelling the mechanical behavior of MWCNT reinforced materials and MWCNT-based complex structures.
- The Young’s and shear moduli of armchair and zigzag MWCNTs are approximately constant with increasing difference between diameters of the outer and inner layers and, consequently, the number of layers. The Young’s and shear moduli of the zigzag structures are lower than for the armchair structures.
- The current values of the Young’s and shear moduli are in good agreement with various results available in the literature, obtained taking into account the van der Waals interactions between layers. This point out an appropriateness of using of the simplified model for evaluation of the elastic properties of the MWCNT structures.

## Figures and Tables

**Table 1:** Input parameters for finite element (FE) simulations of the layers (single-walled carbon nanotubes, SWCNTs) of multiwalled carbon nanotubes: material and geometric properties of beam element.

Parameter	Value	Formulation
Bond stretching force constant, $k_{r}$ [44]	6.52 × 10^−7^ N nm^−1^	–
Bond bending force constant, $k_{\theta }$ [44]	8.76 × 10^−10^ N⋅nm⋅rad^−2^	–
Torsional resistance force constant, $k_{\tau }$ [44,45]	2.78 × 10^−10^ N⋅nm⋅rad^−2^	–
Carbon–carbon (C–C) bond/beam length ($l=a_{C-C}$)	0.1421 nm	–
Diameter (d)	0.147 nm	$d=4\sqrt{k_{\theta }/k_{r}}$
Cross section area, $A_{b}$	0.01688 nm^2^	$A_{b}=\pi d^{2}/4$
Moment of inertia, $I_{b}$	2.269 × 10^−5^ nm^4^	$I_{b}=\pi d^{4}/64$
Polar moment of inertia, $J_{b}$	4.537 × 10^−5^⋅nm^4^	$J_{b}=\pi d^{4}/32$
Young’s modulus, $E_{b}$	5488 GPa	$E_{b}=k_{r}^{2}l/4\pi k_{\theta }$
Shear modulus, $G_{b}$	870.7 GPa	$G_{b}=k_{r}^{2}k_{\tau }l/8\pi k_{\theta }^{2}$
Rigidity, $E_{b}A_{b}$	92.65 nN	$E_{b}A_{b}=k_{r}l$ [24]
Rigidity, $E_{b}I_{b}$	0.1245 nN⋅nm^2^	$E_{b}I_{b}=k_{\theta }l$ [24]
Rigidity, $G_{b}J_{b}$	0.0395 nN⋅nm^2^	$G_{b}J_{b}=k_{\tau }l$ [24]

**Table 2:** Geometrical characteristics of armchair multiwalled carbon nanotubes (MWCNTs), under study.

Interlay. Spacing, $d_{int}[\mathrm{nm}]$	Inner Layer Diameter, $D_{in}$ [nm]	N, Number of Layers		$\left(n_{1},\;m_{1}\right)\ldots \left(n_{N,}m_{N}\right)$	Outer Layer Diameter, $D_{out}$ [nm]
0.339	1.356	2	(10 + 5(N − 1), 10 + 5(N−1)), N = 2 to 20	(10,10) (15,15)	2.034
		3		(10,10) (15,15) (20,20)	2.713
		4		(10,10) (15,15) … (25,25)	3.390
		5		(10,10) (15,15) … (30,30)	4.068
		6		(10,10) (15,15) … (35,35)	4.746
		7		(10,10) (15,15) … (40,40)	5.424
		8		(10,10) (15,15) … (45,45)	6.101
		9		(10,10) (15,15) … (50,50)	6.780
		10		(10,10) (15,15) … (55,55)	7.457
		11		(10,10) (15,15) … (60,60)	8.136
		12		(10,10) (15,15) … (65,65)	8.814
		13		(10,10) (15,15) … (70,70)	9.492
		14		(10,10) (15,15) … (75,75)	10.170
		15		(10,10) (15,15) … (80,80)	10.848
		16		(10,10) (15,15) … (85,85)	11.526
		17		(10,10) (15,15) … (90,90)	12.204
		18		(10,10) (15,15) … (95,95)	12.882
		19		(10,10) (15,15) … (100,100)	13.560
		20		(10,10) (15,15) … (105,105)	14.238
	4.746	2	(35 + 5(N − 1), 35 + 5(N − 1)), N = 2 to 15	(35,35) (40,40)	5.424
		3		(35,35) (40,40) (45,45)	6.102
		4		(35,35) (40,40) … (50,50)	6.780
		5		(35,35) (40,40) … (55,55)	7.458
		6		(35,35) (40,40) … (60,60)	8.136
		7		(35,35) (40,40) … (65,65)	8.814
		8		(35,35) (40,40) … (70,70)	9.492
		9		(35,35) (40,40) … (75,75)	10.170
		10		(35,35) (40,40) … (80,80)	10.848
		11		(35,35) (40,40) … (85,85)	11.526
		12		(35,35) (40,40) … (90,90)	12.204
		13		(35,35) (40,40) … (95,95)	12.882
		14		(35,35) (40,40) … (100,100)	13.560
		15		(35,35) (40,40) … (105,105)	14.238

**Table 3:** Geometrical characteristics of zigzag MWCNTs, under study.

Interlay. Spacing, $d_{int}[\mathrm{nm}]$	Inner Layer Diameter, $D_{in}$ [nm]	N, Number of Layers		$\left(n_{1},0\right)\ldots \left(n_{N,}0\right)$	Outer Layer Diameter, $D_{out}$ [nm]
0.352	1.096	2	(14 + 9(N − 1), 0), N = 2 to 20	(14,0) (23,0)	1.802
		3		(14,0) (23,0) (32,0)	2.507
		4		(14,0) (23,0) … (41,0)	3.212
		5		(14,0) (23,0) … (50,0)	3.916
		6		(14,0) (23,0) … (59,0)	4.618
		7		(14,0) (23,0) … (68,0)	5.323
		8		(14,0) (23,0) … (77,0)	6.027
		9		(14,0) (23,0) … (86,0)	6.732
		10		(14,0) (23,0) … (95,0)	7.436
		11		(14,0) (23,0) … (104,0)	8.142
		12		(14,0) (23,0) … (113,0)	8.847
		13		(14,0) (23,0) … (122,0)	9.551
		14		(14,0) (23,0) … (131,0)	10.256
		15		(14,0) (23,0) … (140,0)	10.960
		16		(14,0) (23,0) … (149,0)	11.665
		17		(14,0) (23,0) … (158,0)	12.370
		18		(14,0) (23,0) … (167,0)	13.074
		19		(14,0) (23,0) … (176,0)	13.779
		20		(14,0) (23,0) … (185,0)	14.483
	4.619	2	(59 + 9(N − 1), 0), N = 2 to 15	(59,0) (68,0)	5.324
		3		(59,0) (68,0) (77,0)	6.028
		4		(59,0) (68,0) … (86,0)	6.733
		5		(59,0) (68,0) … (95,0)	7.437
		6		(59,0) (68,0) … (104,0)	8.142
		7		(59,0) (68,0) … (113,0)	8.847
		8		(59,0) (68,0) … (122,0)	9.551
		9		(59,0) (68,0) … (131,0)	10.256
		10		(59,0) (68,0) … (140,0)	10.960
		11		(59,0) (68,0) … (149,0)	11.665
		12		(59,0) (68,0) … (158,0)	12.370
		13		(59,0) (68,0) … (167,0)	13.074
		14		(59,0) (68,0) … (176,0)	13.779
		15		(59,0) (68,0) … (185,0)	14.483

**Table 4:** Fitting parameters $\alpha_{MW}$, $\beta_{MW}$, and $\gamma_{MW}$ for the armchair and zigzag MWCNTs.

Type of MWCNT	Parameter		
	$\alpha_{MW}$ $\left(nN\cdot nm^{-2}\right)$	$\beta_{MW}$ $\left(nN∙nm^{-2}\right)$	$\gamma_{MW}$ $\left(nN∙nm^{-2}\right)$
Armchair	827.24	52.34	48.02
Zigzag	796.91	50.37	46.44

**Table 5:** Geometrical characteristics of armchair and zigzag MWCNTs for testing the methodology proposed.

MWCNT Type	Inner Layer Diameter, $D_{in}$ [nm]	N, Number of Layers		$\left(n_{1},m_{1}\right)\ldots \left(n_{N,}m_{N}\right)$ $\left(n_{1},0\right)\ldots \left(n_{N,}0\right)$	Outer Layer Diameter, $D_{out}$ [nm]
Armchair $d_{\mathrm{int}}=0.339\;\mathrm{nm}$	1.085	2	(8 + 5(N − 1), 8 + 5(N − 1)), N = 2 to 21	(8,8) (13,13)	1.763
		3		(8,8) (13,13) (18,18)	2.441
		4		(8,8) (13,13) … (23,23)	3.119
		5		(8,8) (13,13) … (28,28)	3.797
		6		(8,8) (13,13) … (33,33)	4.475
		7		(8,8) (13,13) … (38,38)	5.153
		8		(8,8) (13,13) … (43,43)	5.831
		9		(8,8) (13,13) … (48,48)	6.509
		10		(8,8) (13,13) … (53,53)	7.187
		11		(8,8) (13,13) … (58,58)	7.865
		12		(8,8) (13,13) … (63,63)	8.543
		13		(8,8) (13,13) … (68,68)	9.221
		14		(8,8) (13,13) … (73,73)	9.899
		15		(8,8) (13,13) … (78,78)	10.577
		16		(8,8) (13,13) … (83,83)	11.255
		17		(8,8) (13,13) … (88,88)	11.933
		18		(8,8) (13,13) … (93,93)	12.611
		19		(8,8) (13,13) … (98,98)	13.289
		20		(8,8) (13,13) … (103,103)	13.967
		21		(8,8) (13,13) … (108,108)	14.645
Zigzag $d_{\mathrm{int}}=0.352\;\mathrm{nm}$	0.783	2	(10 + 9(N − 1), 0), N = 2 to 21	(10,0) (19,0)	1.487
		3		(10,0) (19,0) (28,0)	2.192
		4		(10,0) (19,0) … (37,0)	2.897
		5		(10,0) (19,0) … (46,0)	3.601
		6		(10,0) (19,0) … (55,0)	4.306
		7		(10,0) (19,0) … (64,0)	5.010
		8		(10,0) (19,0) … (73,0)	5.715
		9		(10,0) (19,0) … (82,0)	6.420
		10		(10,0) (19,0) … (91,0)	7.124
		11		(10,0) (19,0) … (100,0)	7.829
		12		(10,0) (19,0) … (109,0)	8.533
		13		(10,0) (19,0) … (118,0)	9.238
		14		(10,0) (19,0) … (127,0)	9.943
		15		(10,0) (19,0) … (136,0)	10.647
		16		(10,0) (19,0) … (145,0)	11.352
		17		(10,0) (19,0) … (154,0)	12.056
		18		(10,0) (19,0) … (163,0)	12.761
		19		(10,0) (19,0) … (172,0)	13.466
		20		(10,0) (19,0) … (181,0)	14.170
		21		(10,0) (19,0) … (190,0)	14.875

**Table 6:** Comparison of the current results of the elastic moduli results with those reported in the literature.

Reference	Method	Interlayer Spacing, $d_{int},\;\mathrm{nm}$	Approach for the vdW Interactions between Layers	MWCNT Type *	Max. Number of Layers, N	Young’s Modulus, TPa	Shear Modulus, TPa
Hwang et al. [21]	MD: empirical Tersoff three-body potential	0.339	Empirical Tersoff three-body potential	Armchair (5,5)(10,10), (10,10)(15,15)	2	0.85–1.16	–
				Armchair (5,5)(10,10)(15,15)	3	0.85–1.12	
Santosh et al. [22]	MD: force- constant approach	0.340	Force-constant approach	Armchair (5,5)(10,10), (7,7)(12,12), (10,10)(15,15), (15,15)(20,20), (20,20)(25,25)	2	1.02	0.27
Tu and Ou-Yang [23]	CM: classic shell theory	0.340	–	–	100	1.70–1.05	–
Li and Chou [24]	NCM: beams	0.339	Truss rods	Armchair (3,3)(8,8)(13,13)(18,18)	4	1.05–1.10	0.33–0.48
		0.352		Zigzag (5,0)(14,0)(23,0)(32,0)		1.05–1.12	0.33–0.36
Kalamkarov et al. [25]		0.339	Springs	Armchair (5,5)(10,10)(15,15)(20,20)	4	1.00–1.45	0.44–0.47
		0.352		Zigzag (5, 0)(14, 0)(23, 0)(32, 0)		0.96–1.50	0.44–0.47
Fan et al. [30]		0.352	Springs: interlayer pressure	Zigzag (5,0)(14,0)(23,0)	3	1.007–1.011	0.430–0.398
				(12,0)(21, 0)(30,0)		1.021–1.017	0.415–0.347
				(15,0)(24, 0)(33,0)		1.023–1.018	0.402–0.347
Nahas and Abd-Rabou [31]		–	Beams	Armchair	3	0.98–1.02	–
				Zigzag		0.876–0.937	
Ghavamian et al. [27]	NCM: beams	0.339	Springs	Armchair (10,10)(15,15)…(30,30)	5	1.040–1.044	–
		0.352		Zigzag (14,0)(23,0)…(50,0)		1.030–1.035	
Ghavamian et al. [28]		0.339	Springs	Armchair (10,10)(15,15)…(30,30)	5	–	0.50
		0.352		Zigzag (14,0)(23,0)…(50,0)			
Almagableh et al. [32]	NCM: pseudo-rectangular beams	0.340	Nonlinear solid elements	Zigzag (5,0)(14,0), (9,0)(18,0), (12,0)(21,0), (21,0)(30,0)	2	0.65–0.2	–
Treacy et al. [46]	TEM: amplitude of intrinsic thermal vibrations	–	–	Unidentified type	–	1.8 ± 0.9	–
Kashyap and Patil [47]	TEM image +analytical	–	–	Unidentified type	–	0.9	
Current results	NCM: beams	0.339	–	Armchair (10,10)(15,15)…(105,105)	20	1.061–1.051	0.479–0.484
		0.352		Zigzag (14,0) (23,0)…(185,0)		1.069–1.012	0.475–0.471

* The MWCNTs were produced starting from the smallest diameter layer (indicated in the table in the initial position) by adding subsequent layers until maximum number of layers.
